# Analysis on the Incidence of the Fibularis Quartus Muscle Using Magnetic Resonance Imaging

**DOI:** 10.1155/2012/485149

**Published:** 2012-07-09

**Authors:** Sérgio Ricardo Rios Nascimento, Renata Watanabe Costa, Cristiane Regina Ruiz, Nader Wafae

**Affiliations:** ^1^Macroscopic Anatomy and Anatomy by Radiologic Imaging, São Camilo University Center, São Paulo, SP, Brazil; ^2^Biomedicine, São Camilo University Center, São Paulo, SP, Brazil; ^3^São Camilo University Center, 04263-100 São Paulo, SP, Brazil; ^4^Paulista School of Medicine, Federal University of São Paulo, Rua Botucatu, 740, V. Clementino, 04023-062 São Paulo, SP, Brazil

## Abstract

*Objective*. Quantify the presence of the fibularis quartus muscle and correlate it with the individual's sex and concomitant presence of the fibularis tertius muscle. *Materials and Methods*. We evaluated 211 magnetic resonance examinations (43.13% men and 56.87% women) on the ankle and hindfoot, produced between 2009 and 2011. *Results*. The fourth fibularis muscle was found to be present in 7.62% of the examinations and 75% of these also contained the fibularis tertius muscle. *Conclusion*. The incidence of the fourth fibularis muscle is variable; moreover, its incidence does not depend on the individual's gender or the presence of the fibularis tertius muscle.

## 1. Introduction

The fibularis quartus muscle was first described by Otto in 1816 [1] and was subsequently studied in detail by Hecker in 1923 [2]. It forms part of one of the three groups of muscle variations that occur in the ankle: the group of muscle-tendon variants of the fibular muscles [3]. In most cases, the presence of this muscle is asymptomatic, and it is detected by chance during the examination or surgical procedure [4–6]. However, in some cases, its presence is associated with certain symptoms: pain in the ankle, with or without anterior trauma; displacement, spraining, or tearing of the fibularis brevis tendon; tendon calcification; or painful hypertrophy of the retrotrochlear eminence [4, 5, 7]. The presence of the fibularis quartus muscle may simulate a longitudinal tear in the fibularis brevis tendon, but is differentiated by the presence of the muscle, when a muscle belly separated from the muscle belly of the fibularis brevis is present [8].

In most cases, the origin of the fibularis quartus muscle is posterior to the bifurcation of the belly of the fibularis muscles, or in the fibularis brevis muscle. It may also originate in the posterior dace of the fibula or in the fibularis longus muscle. Its insertion is also variable, thus explaining the variety of names that it has received: accessory fibularis muscle (insertion into the tendon of the fibularis longus, in the sole of the foot); fibulocalcaneus muscle (insertion into the retrotrochlear eminence); fibulocuboid muscle (insertion into the tuberosity of the cuboid bone, inferiorly); or fibular-fibularis longus muscle (insertion into the tendon of the fibularis longus muscle or into the inferior retinaculum of the fibularis muscles, adjacent to the retrotrochlear eminence). These last two variants are the ones with lowest incidence [6, 9, 10]. The fibularis quartus muscle acts predominantly as a pronator for the foot, as do the other fibularis muscles (longus and brevis). It has been successfully used in surgical procedures to repair and reconstruct the retinaculum of the fibularis muscles, in treating subluxation of the tendons of the fibularis muscles [6, 11].

Studies on the incidence of the fibularis quartus muscle have presented greatly varying results, along with greatly varying nomenclature. Studies on cadavers have presented incidence rates ranging from 13% to 23%. Using ultrasound, it was found to be present in 22% of the sample. Using magnetic resonance imaging (MRI), it was found in 10% of the examinations [6].

Its absence in prosimians and simians and presence in humans led Hecker, in 1923 [2], to believe that this muscle was an indication of evolution or adaptation to the environment, given that its main function is to raise the lateral edge of the foot and stabilize its pronation, which are important for evolution and maintenance of bipedal posture [9]. The presence of this muscle, along with the presence of the fibularis tertius muscle, constitutes a strong indication of anatomical adaptation of the human ankle to the bipedal condition, thereby contributing towards stabilization and reinforcement of this important joint. This suggests that the symptoms associated with its presence are an indication that this adaptation has not yet completed its evolution.

Therefore, given the anatomical and clinical importance of this muscle, our aim in this study was to quantify the presence of the fibularis quartus muscle and correlate its incidence with the individual's sex and concomitant presence of the fibularis tertius muscle.

## 2. Methodology

We evaluated 211 MRI examinations on the ankle or hindfoot between 2009 and 2011. All the examinations were performed using the Signa Horizon Lx machine (General Electric Medical Systems), with a 1.5 T magnetic field and a quadrature coil for knees. The images analyzed were acquired using the spin echo technique with T1 weighting, without administering contrast medium. The images were acquired in three planes: axial—programmed parallel to the plantar fascia, from the calcaneus; sagittal—programmed parallel to the plane of the tibia, perpendicular to the sole of the foot; coronal—programmed perpendicular to the tibial-calcaneal joint. The examinations used came from the database of the imaging diagnostics center of Hospital Santa Catarina (stored on CD-R) and were used in accordance with the authorization from the institution's ethics committee. The data were analyzed using the ONIS 2.3 Free Edition software (Digitalcore), which is available from http://www.onis-viewer.com/, with the aid of the hospital's radiologists. The patient's identities were kept absolutely confidential, and only the patient's gender and the side of the ankle examined were recorded.

The exclusion criteria were that examinations showing evident muscle and/or ligament injuries, or tumor lesions of any nature, and examinations performed on patients after surgical procedures, were rejected.

In assessing whether the fibularis quartus muscle was present, the following reference points were taken into consideration. In the axial plane, made with 4 mm of thickness and 1 mm of gap, observations were made starting from the slice in which the two malleoli appeared. The entire extent of the fibularis muscles and their divisions into the fibularis longus and brevis was examined, while remaining attentive for the possible presence of a third division that would suggest the presence of the fibularis quartus muscle. The slices were examined sequentially from the uppermost slice considered to the calcaneus bone, and the insertion of this third division of the fibularis muscles was examined to determine whether the insertion was into the fibular trochlea of the calcaneus, which would characterize presence of the fibularis quartus muscle.

In the sagittal plane, made with 3 mm of thickness and 1 mm of gap, in the lateral-medial direction, the presence of a third muscle belly was observed just after the appearance of the muscle bellies of the fibularis longus and fibularis brevis. If the fibularis quartus muscle was present, it would be observed in a slice in which the fibula bone could no longer be seen, that is, close to the talus-calcaneus joint. 

In the coronal plane, made with 4 mm of thickness and 1 mm of gap, slices in the region of the fibular trochlea of the calcaneus were analyzed. There, the tendons of the fibularis longus and brevis muscles were observed, along with a third tendon inserted into the fibular trochlea, thus suggesting the presence of the fibularis quartus muscle [12].

## 3. Results

Of the 211 tests evaluated, 91 (43.13%) were men, 44 (48.35%) on the right side and 47 (51.65%) on the left side, and 120 (56.87%) were women, 56 (46.67%) on the right side 64 (53.33%) on the left. Ankles rights total 100 (47.39%) and 111 left ankle (52.61%).

**Figure 1:**
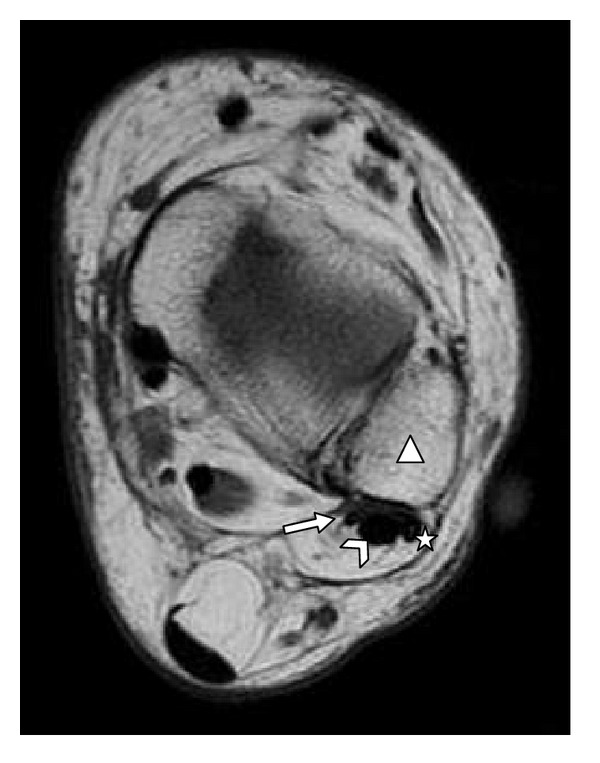
MRI, transverse T1 18/660 of left ankle. Triangle: left fibula; star: fibularis longus tendon; arrowhead: fibularis brevis tendon; arrow: fibularis quartus tendon.

**Figure 2:**
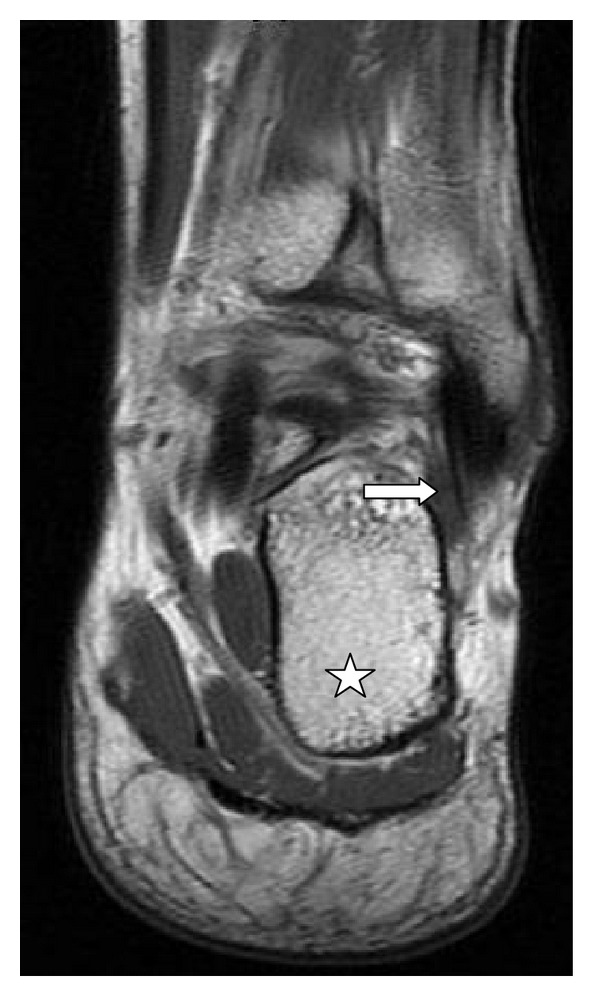
MRI, coronal T1 18/660 of left ankle. Star: calcaneus bone; arrow: fibularis quartus muscle.

**Figure 3:**
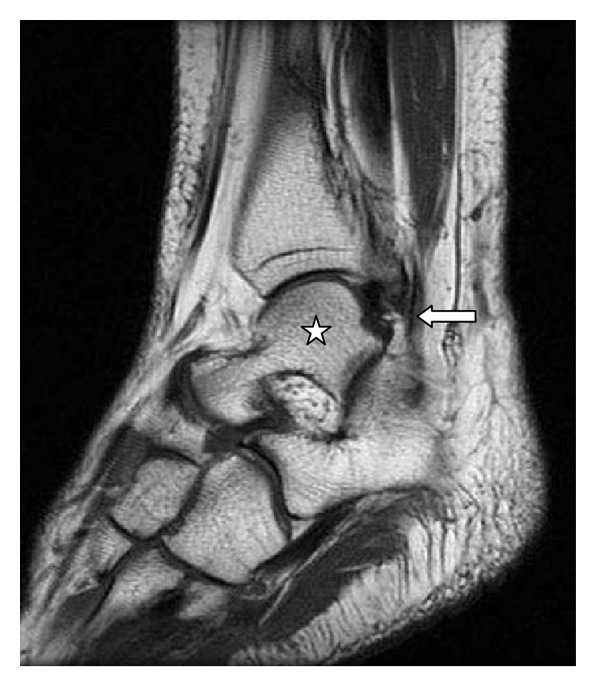
MRI, sagittal T1 23/550 of left ankle. Star: talus bone; arrow: fibularis quartus muscle.

The fibularis quartus muscle was observed in 16 ankles (7.62%) (Figures 1, 2, and 3), seven women (43.75%) and 9 male (56.25%) (Table 1). Overall, it was observed the presence of muscle in 5 right ankles (31.25%) and 11 left ankles (68.75%).

In ankles containing the fibularis quartus muscle, 12 (75%) also contained the fibularis tertius muscle, 6 males and 6 women ankles (Table 2).

Based on these results was then carried out a cross-sectional study, distributed in 2 × 2 tables (Tables 1 and 2). For the results presented in Table 1 were performed using chi-square test, *t*-student and the coefficients of Fi and Yule considering all *α* = 0.05 [13, 14]. For the results presented in Table 2 was used Fisher's test and the coefficient of Yule.

According to data obtained in this study—*χ* ^2^ = 1,2154; *t* = 1,1004; *φ* = 0,0759; *q* = 0,278—at the 5% level of significance, the degree of association between the presence of fibularis quartus muscle and the individual's gender is low and relatively significant, and therefore we cannot reject the hypothesis that the presence of fibularis quartus muscle is not associated with the individual's gender.

The degree of association between the presence of fibularis quartus muscle along with the fibularis tertius muscle and gender is also low and not very significant; therefore we cannot reject the hypothesis that the individual's gender and the presence of the muscles are independent variables—*P* = 0,3923, *q* = 0.5. In cases where there is the presence of both, the ratio between the muscles presence in men and women is the same.

## 4. Discussion

Since its discovery in 1826, the fibularis quartus muscle has been objective of researches with different results, but complementaries (Tables 3 and 4).

The fibularis quartus muscle is described as a variation of the fibularis brevis muscle in the majority of the authors. Goss [10] states that the most common incidence is as fibulocalcaneus externum, originating at the back of fibula between the fibularis brevis and flexor hallucis longus with insertion on the fibular trochlea, as a statistical citing Gruber's work of dissection [15] with 3% of incidence, although both authors do not disclose the size of the sample. Standring [16] only cited as one variation of the fibularis brevis muscle, assuming that its insertion can vary and may be in the calcaneus or in the cuboid.

Le Double [23] however, states that the fibularis quartus muscle, when fully developed, has origins in the lateral distal quarter of fibula below the fibularis brevis muscle, followed by malleolar sulcus, inserting on the base, on the proximal phalanx, or middle phalanx of fifth toe. Also according to Le Double [23] the presence of fully developed fibularis quartus muscle is quite rare and usually has its distal end withered, and this would be the reason why this muscle receives various names, according to the author who first described: Fibulocuboid muscle—Chudzinski; Fibularis accessorius muscle—Henle; Fibulocalcaneus externum muscle—Wood, Theile, and Macalister; Fibularis quartus muscle—Otto; Fibulofibularis accessorius sixth or lower—Macalister. However, Testut [24] considers the muscles fibulocuboid and fibulocalcaneus externum as a variant of fibularis quartus, and this as an incomplete variable of the fifth finger fibularis muscle. Poirier and Charpy [25] consider it as one of the many incomplete variants of the muscle known as fifth finger fibularis muscle, citing although this muscle is also present in chimpanzees and in a number of mammals.

Testut and Latarjet [26] treat any supernumerary tendon of the fibularis brevis muscle, with or without muscle belly, which has no inferior insertion at the base of the fifth finger as a rudimentary and atrophied fifth finger fibularis muscle.

Testut [24] explains that the absorption of this muscle by the others fibularis muscles—brevis and longus—would be something normal and expected due to its low incidence, investigated by Wood [17, 18] and Pozzi [19] (Table 3). Le Double [23] reinforces this theory by explaining that, unlike the upper limbs, where the muscle movements are more diverse and accurate, the muscular action of the lower limbs comes down to support body weight, balance, and gait, and therefore the lateral muscles of the lower limb, which have same function and action, are in the process of absorption by the fibularis brevis and longus muscles, larger and more constants.

Its incidence varies among studies conducted after the nineteenth century (Table 4), from 0% to 26% [3–6, 9, 11, 20, 21].

The Testut [24] and Le Double [23] theory is based on the low incidence of fibularis quartus muscle, suggesting that this muscle would be absorbed by other fibularis muscles and disappear however, the incidence of fibularis quartus muscle remains, and the new analytical methods—ultrasound and MRI—showed its incidence to be increasing, although this can be explained by the increase of the studied sample and easy assessment of this muscle by the new imaging methods already mentioned.

It is useful to highlight that with the advancement of diagnostic imaging techniques, particularly magnetic resonance imaging, its study and evaluation are now simpler and more accurate, but not less intriguing. Its incidence varies according to the method used for research, with lower prevalence observed in studies using cadavers and higher incidence in studies using ultrasound exams [6]. Our results fall within the limits of what has been demonstrated until now by several studies conducted on it which suggests that after more than 180 years of its first description, its incidence is still imprecise and inconsistent.

His presence, even in a few cases, is associated with some diseases that affect the ankle joint and may also be a differential diagnosis for the hypothesis of longitudinal rupture of the fibularis brevis. However, its presence has providential value in cases where surgical repair or reconstruction of the inferior fibularis retinaculum is necessary, as described by Mick and Lynch [27] as “[*⋯*] an excellent source of tissue for reconstruction of the peroneal retinaculum to prevent recurrent dislocation of the peroneal tendons.”

The presence of this muscle together with the fibularis tertius muscle—already well established, with 83–95% of incidence [6, 28]—is contributing to the possibility of human adaptation at bipedal gait, composing a muscle tendon not only stabilizer of the ankle joint, but also contributing effectively to save energy during walking, working in eversion (fibularis quartus muscle), and dorsiflexion (fibularis tertius muscle) of the foot during the bipedal swing gait [16]. The absence of the fibularis tertius muscle—considering an evolutionary muscle to be uniquely human and its importance in bipedal gait—is still discussed by some authors, about the lateral support to the foot during gait would be compromised [29, 30].

Other authors investigated [31–38] did not mention the fibularis quartus muscle, whether as a variation or as incomplete or stunted form of another muscle. These authors cite only the presence of fibularis tertius muscle as a constant and not as an inconstant muscle.

Our results indicate that its incidence is independent of the individual's gender, being common to human beings as a whole, suggesting no relationship to gender.

Still according to the results obtained, the chance that a person who has the fibularis quartus muscle also has fibularis tertius muscle is three to one; in other words, one of three individuals that have the fibularis quartus muscle also has a fibularis tertius muscle, whether man or woman. Therefore, the presence of fibularis tertius muscle does not depend on the presence of the fibularis quartus muscle in contrast, the presence of fibularis quartus muscle increases the chances that there is a presence of fibularis tertius muscle too.

## 5. Conclusions

The fibularis quartus muscle was found in 7.62% of the population. In regarding to the side, its incidence was higher in left ankle (68.79%) than in rights (31.25%). In regarding to the fibularis tertius muscle, 75% of the ankles containing the fibularis quartus muscle also had this muscle. The incidence of fibularis quartus muscle, together with the fibularis tertius muscle remains inconstant, variable, and independent of the individual's gender as well as the presence of the fibularis tertius muscle. However, the chance of a person who has fibularis tertius muscle is three times bigger when the fibularis quartus muscle is present. The presence of fibularis quartus muscle is asymptomatic in most cases, but its presence is an important source of tissue for reconstruction procedures of fibular retinaculum, must be evaluated carefully, and be considered as a differential diagnosis in cases of suspect of longitudinal rupture of fibular brevis tendon.

## Figures and Tables

**Table 1:** Incidence of the fibularis quartus muscle.

	Absent	Present	Total
Female	113	7	120
Male	82	9	91

Total	195	16	211

**Table 2:** Incidence of the fibularis quartus muscle together with the fibularis tertius muscle.

	Male	Female	Total
Fibularis 4th	3	1	4
Fibularis 3rd and 4th	6	6	12

Total	9	7	16

**Table 3:** Quantitative research on the incidence of fibularis quartus muscle, XIX century, by the method of dissection in cadavers.

Author	Year	Method	*n*	Incidence	%
Wood [17]	1866	Cadavers	32	5	15.6%
Wood [18]	1867	Cadavers	70	2	2.9%
Pozzi [19]	1872	Cadavers	28	4	14.3%

Total			130	11	8.5%

**Table 4:** Quantitative research on the incidence of fibularis quartus muscle, XX and XXI centuries, by different methods.

Author	Year	Method	*n*	Incidence	%
Sobel et al. [20]	1990	Cadavers	124	27	21.8%
Cheung et al. [9]	1997	MRI	136	14	10.3%
Rosenberg et al. [21]	1997	MRI	41	2	4.9%
Chepuri et al. [11]	2001	Ultrasound/MRI	32	7	21.9%
Borne et al. [4]	2002	MRI	63	7	11.1%
Zammit and Singh [3]	2003	Cadavers	102	6	5.9%
Zammit and Singh [3]	2003	MRI	80	6	7.5%
Saupe et al. [5]	2007	MRI	65	11	16.9%
Tubbs et al. [22]	2008	Cadavers	88	0	0.0%

Total			731	80	10.94%
